# Survival in untreated hepatocellular carcinoma: A national cohort study

**DOI:** 10.1371/journal.pone.0246143

**Published:** 2021-02-04

**Authors:** Young Ae Kim, Danbee Kang, Hyeyoung Moon, Donghyun Sinn, Minwoong Kang, Sang Myung Woo, Yoon Jung Chang, Boram Park, Sun-Young Kong, Eliseo Guallar, Soo-Yong Shin, Geunyeon Gwak, Joung Hwan Back, Eun Sook Lee, Juhee Cho

**Affiliations:** 1 Division of Cancer Control & Policy, National Cancer Control Institute, National Cancer Center, Goyang, South Korea; 2 Department of Clinical Research Design and Evaluation, SAIHST, Sungkyunkwan University, Seoul, South Korea; 3 Center for Clinical Epidemiology, Samsung Medical Center, Sungkyunkwan University School of Medicine, Seoul, South Korea; 4 Department of Medicine, Samsung Medical Center, Sungkyunkwan University School of Medicine, Seoul, South Korea; 5 Department of Digital Health, SAIHST, Sungkyunkwan University, Seoul, South Korea; 6 Center for Liver and Pancreatobiliary Cancer, Research Institute, National Cancer Center, Goyang, Korea; 7 Biostatistics Collaboration Team, Research Core Center, Research Institute, National Cancer Center, Goyang, South Korea; 8 Division of Translational Science, Research Institute, National Cancer Center, Goyang, South Korea; 9 Department of Epidemiology, and Welch Center for Epidemiology, Prevention, and Clinical Research, Johns Hopkins Bloomberg School of Public Health, Baltimore, Maryland, United States of America; 10 Health Insurance Policy Research Institute, National Health Insurance Service, Gangwon-do, Korea; 11 Department of Surgery, Research Institute & Hospital, National Cancer Center, Goyang, South Korea; Texas A&M University, UNITED STATES

## Abstract

This study aimed to analyze the proportion, characteristics and prognosis of untreated hepatocellular carcinoma (HCC) patients in a large representative nationwide study. A cohort study was conducted using the National Health Insurance Service (NHIS) database in Korea. A total of 63,668 newly-diagnosed HCC patients between January 2008 and December 2013 were analyzed. Patients were categorized into treatment group and no treatment group using claim codes after HCC diagnosis. The proportion of untreated HCC patients was 27.6%, decreasing from 33.4% in 2008 to 24.8% in 2013. Compared to treated patients, untreated patients were more likely to be older (*P* < 0.001), female (*P* < 0.01), to have a distant SEER stage (*P* < 0.001), severe liver disease (*P* < 0.001), and lower income (*P* < 0.001). The fully-adjusted hazard ratio for all-cause mortality comparing untreated to treated patients was 3.11 (95% CI, 3.04–3.18). The risk of mortality was higher for untreated patients in all pre-defined subgroups, including those with distant SEER stage and those with severe liver disease. About one fourth of newly diagnosed HCC patients did not receive any HCC-specific treatment. Untreated patients showed higher risk of mortality compared to treated patients in all subgroups. Further studies are needed to identify obstacles for HCC treatment and to improve treatment rates.

## Introduction

Primary liver cancer, mostly hepatocellular carcinoma (HCC), is the sixth most common cancer and the second most common cause of cancer mortality in the world [1–3]. While the prognosis of HCC is dismal, due in large part to late detection and to compromised liver function by the underlying chronic liver disease [4–6], recent advances in HCC management include several potentially efficacious treatments for HCC [5, 7]. In addition, modern antiviral agents induce sustained suppression of viral replication or viral eradication in most patients with chronic hepatitis B virus or hepatitis C virus infection [8, 9], improving or maintaining liver function [10] and allowing for the application of HCC treatments. In spite of these advances, a substantial number of HCC patients still do not receive HCC-specific treatment. In an analysis of 128 hospitals from the US Veterans Administration, 24% of HCC patients did not receive HCC-specific treatment [11], and over 60% of HCC patients in the US Surveillance, Epidemiology, and End Results (SEER)-Medicare database did not receive any HCC-specific treatment [12]. Similarly, in the Italian Liver Cancer (ITA.LI.CA) database from 21 medical institutions, 11.7% of patients did not receive HCC-specific treatment [13].

Advanced stage at diagnosis, end-stage liver disease, old age and comorbidities have been suggested as possible reasons for not receiving treatment [12–15]. However, even patients with early-stage HCC and no co-morbidities may receive no treatment due to uncertain reasons [13, 14]. So far, the characteristics and clinical implications of untreated HCC have not been assessed in a comprehensive national analysis. In this study, we used a national cohort database to evaluate the proportion, characteristics, and clinical outcome of untreated HCC.

## Materials and methods

### Study population and design

We conducted a retrospective nationwide cohort analysis of newly diagnosed HCC patients 18 to 80 years of age between January 1^st^, 2008 and December 31^st^, 2013 (N = 68,558). We then excluded patients who had a history of any type of cancer (N = 3,973) and those who were diagnosed at the time of death (N = 1,022). The final sample size was 63,668 (50,963 men and 12,705 women). The Institutional Review Board of the Korean National Cancer Center approved this study and waived the requirement for informed consent because of the retrospective nature of our study using de-identified data.

### Data sources

In this study, we used three sources of data. Cancer-related information was collected using the Korea National Cancer Incidence (KNCI) database, a nationwide hospital-based cancer registry that collects information from all cancer cases in Korea since 1999 [16]. The KNCI database contains information on age, sex, residential area, date of diagnosis, primary cancer site, and SEER stage of the primary tumor.

Information on treatments and diagnostic procedures, including details of diseases and prescriptions, was obtained from claims data from the Korean National Health Insurance Service (NHIS) database [17]. The NHIS is the public single payer for Korea’s mandatory universal medical insurance system. To claim reimbursement for patient care, all clinics and hospitals in Korea submit data to NHIS on inpatient hospitalizations and outpatient visits, allowing follow-up of the entire healthcare service utilization of any given patient over the course of treatment [18]. This information includes diagnoses, prescriptions, and medical procedures, which are coded using ICD-10, International Classification of Diseases, Tenth Revision (ICD-10) codes and Korean Drug and Anatomical Therapeutic Chemical (ATC) Codes. NHIS routinely audits the claims, and the data are considered reliable and have been used in numerous peer-reviewed publications [17, 19]. The NHIS database comprises four databases on insurance eligibility, medical treatments, medical care institutions, and general health exams. The medical treatment database contains information from treatment bills, including details of diseases and prescriptions [20]. Finally, vital status through December 31^st^, 2014 was ascertained from mortality and population registration data from the Ministry of the Interior.

### Study variables

Our analysis compared patients who received vs. those who did not receive any HCC-specific anticancer treatment. HCC anticancer treatments included radiofrequency ablation (RFA), surgical resection, liver transplantation, transarterial chemoembolization (TACE), target drugs, radiation therapy, and other HCC-specific treatments. Treatments were identified by procedure codes and Korean ATC codes. If a patient did not receive any HCC-specific treatment after HCC diagnosis, we considered the patient untreated.

Potential confounders included age, sex, year of diagnosis, SEER tumor stage, severity of liver disease, and income percentile. HCC tumor stage was classified using SEER staging as localized (limited to the liver), regional (tumor extension beyond the limits of the liver), distant (away from the primary tumor), and unknown (lacking sufficient information to assign a stage). For each HCC patient, we assigned the likely cause of chronic liver disease based on diagnostic codes in claims during the overall study period. Hepatitis C was defined as the presence of a code for chronic hepatitis C only (B18.2). Hepatitis B was defined as the presence of a code for chronic hepatitis B (B18.0, B18.1, B18.10, B18.18, or Z22.5). The etiology of chronic liver disease in patients not meeting any of the above criteria was defined as other.

Severity of liver disease was defined based on diagnostic codes in claims during the year prior to HCC diagnosis. Severe liver disease was defined as the presence of a code for alcoholic hepatic failure (k704), hepatic failure (K720, K721, K729), hepatorenal syndrome (k729), esophageal varices with bleeding (I850), jaundice (R17), ascites (R18), hematemesis (K920), melena, hematochezia (K921), hepatopulmonary syndrome (K768), or spontaneous bacterial peritonitis (K658).

We also considered the following co-morbidities that could severely reduce life expectancy diagnosed during the year prior to HCC diagnosis: myocardial infarction (I21, I22), congestive heart failure (I43, I50, I09.9, I11.0, I13.0, I13.2, I25.5, I42.0, I42.5–I42.9, P29.0), cerebrovascular disease (G45, G46, I60–I69, H34.0), dementia (F00–F03, F05.1, G30, G31.1), renal disease (N18, N19, N05.2 –N05.7, N25.0, I12.0, I13.1, N03.2 –N03.7, Z49.0 –Z49.2, Z94.0, Z99.2), paraplegia and hemiplegia (G81, G82, G04.1, G11.4, G80.0, G83.0, G83.1–G83.4,G83.9), diabetes mellitus with complication (E10.2–E10.5, E10.7, E11.2–E11.5, E11.7, E12.2–E12.5, E12.7, E13.2–E13.5, E13.7, E14.2–E14.5, E14.7), and AIDS/HIV (B20–B22, B24).

### Statistical analysis

We used logistic regression to identify factors associated with no treatment. The primary outcome of this study was all-cause mortality and the secondary outcome was liver-specific mortality (ICD-10 code C22). Person-time was calculated from the date of HCC diagnosis to death or the end of the study period on December 31^st^, 2014. Survival curves were generated by the Kaplan-Meier product-limit method and compared by log-rank tests. We used Cox proportional hazards regression models to estimate hazard ratios (HRs) with 95% confidence intervals (CIs) for all-cause mortality comparing treated versus untreated HCC patients. Since treatment decisions and patient survival could be clustered by hospital, we used hospital as a stratification factor in Cox models. We adjusted for sex, age, year of diagnosis, SEER stage, severe liver disease, residential area (urban vs rural), and income percentiles. We examined the proportional hazards assumption using plots of the log(-log) survival function and Schoenfeld residuals.

We performed subgroup analyses to evaluate if the association of treatment status with mortality varied across pre-specified subgroups defined by age (< 50, 50–60, 60–70, and ≥70 years), sex, SEER stage, comorbidities, etiology (hepatitis B only or combined with hepatitis C virus, hepatitis C virus only, and other), severe liver disease, income percentile (Medical aid beneficiary, ≤ 30^th^, > 30^th^– ≤ 70^th^, > 70^th^) and residential area (urban vs rural). We considered a *p*-value of < 0.05 as statistically significant. All analyses were performed using STATA version 14 (StataCorp LP, College Station, TX, USA) and SAS 9.4 (SAS Institute, Cary, NC, USA).

## Results

The median (interquartile range) age at HCC diagnosis of study participants (N = 63,668) was 59 (51–68) years and 80.0% of participants were men. The proportion of untreated patients was 27.6%, and this proportion decreased progressively from 33.4% in 2008 to 24.8% in 2013. Untreated patients were more likely to be older, female, have a distant SEER stage, have comorbidities that could severely reduce life expectancy, have severe liver disease, have lower income and live in a rural area compared to treated patients (Table 1). In multivariable analysis, older age, female, distant SEER stage, comorbidity, severe liver disease, being a Medical aid beneficiary, and living in a rural area were significantly associated with an increased probability of not getting treatment (Table 2).

**Table 1:** Characteristics of study participants.

	Untreated	Treated	*P*-value
	(N = 17,569)	(N = 46,099)	
	N (%)	N (%)	
**Age** (years), median (IQR)	61 (52–71)	58 (51–66)	<0.001
**Age categories**			
<50	3,161 (18.0)	9,214 (20.0)	
50≤-<60	4,875 (27.8)	16,430 (35.6)	
60≤-<70	4,379 (24.9)	13,085 (28.4)	
>70	5,154 (29.3)	7,370 (16.0)	
**Sex**			< 0.01
Male	13,908 (79.2)	37,055 (80.4)	
Female	3,661 (20.8)	9,044 (19.6)	
**Year of Diagnosis**			<0.001
2008	3,467 (19.7)	6,915 (15.0)	
2009	3,151 (17.9)	7,607 (16.5)	
2010	3,126 (17.8)	7,774 (16.9)	
2011	2,693 (15.3)	8,093 (17.6)	
2012	2,560 (14.6)	7,921 (17.2)	
2013	2,572 (14.6)	7,789 (16.9)	
**Seer Stage**			<0.001
Localized	6,169 (35.1)	26,999 (58.6)	
Regional	4,669 (26.6)	10,285 (22.3)	
Distant	3,409 (19.4)	4,014 (8.7)	
Unknown	3,322 (18.9)	4,801 (10.4)	
**Comorbidity**			
Myocardial infarction	131 (0.8)	292 (0.6)	0.12
Congestive heart failure	693 (3.9)	1,167 (2.5)	<0.001
Cerebrovascular disease	1,341 (7.6)	2,289 (5.0)	<0.001
Dementia	487 (2.8)	428 (0.9)	<0.001
Renal disease	328 (1.9)	625 (1.4)	<0.001
Paraplegia and hemiplegia	188 (1.1)	225 (0.5)	<0.001
Diabetes mellitus with complication	2,059 (11.7)	4,674 (10.1)	<0.001
AIDS/HIV.	7 (0.0)	14 (0.0)	0.56
**Severe liver disease, yes**	2,572 (14.6)	4,624 (10.0)	<0.001
**Etiology**			<0.001
Hepatitis B virus*	8,288 (47.2)	33,465 (72.6)	
Hepatitis C virus	1,195 (6.8)	3,683 (8.0)	
Other†	8,086 (46.0)	8,951 (19.4)	
**Income percentile**			<0.001
Medical aid beneficiary	2,991 (17.0)	5,861 (12.7)	
≤30	4,328 (24.6)	11,032 (23.9)	
31–70	5,387 (30.7)	14,323 (31.1)	
>70	4,863 (27.7)	14,883 (32.3)	
**Residency area**			<0.001
Urban	10,627 (60.5)	29,308 (63.6)	
Rural	6,675 (38.0)	16,116 (35.0)	
Unknown	267 (1.5)	675 (1.5)	

Values were presented n (%) or median (IQR)

* Including patients co-infected with HBV and HCV

^†^ Including patients who had other liver disease except HBV and HCV (cirrhosis, steatohepatitis, and fatty liver etc.) or unknown.

**Table 2:** Odds ratios (95% confidence interval) for factors associated with no treatment in patients with newly diagnosed hepatocellular carcinoma.

	Univariable	Multivariable
	OR (95% CI)	OR (95% CI)
**Age**	1.03 (1.02, 1.03)	1.01 (1.01, 1.02)
**Sex**		
Male	*Reference*	*Reference*
Female	1.08 (1.03, 1.13)	1.14 (1.09, 1.20)
**Year of Diagnosis**		
2008	1.52 (1.43, 1.61)	1.58 (1.48, 1.68)
2009	1.25 (1.18, 1.33)	1.27 (1.19, 1.36)
2010	1.22 (1.15, 1.29)	1.24 (1.16, 1.36)
2011	1.01 (0.95, 1.07)	1.06 (0.99, 1.13)
2012	0.98 (0.92, 1.04)	0.99 (0.93, 1.06)
2013	*Reference*	*Reference*
**Seer Stage**		
Localized	*Reference*	*Reference*
Regional	1.99 (1.90, 2.08)	2.06 (1.97, 2.16)
Distant	3.72 (3.52, 3.92)	3.67 (3.46, 3.88)
Unknown	3.03 (2.87, 3.19)	2.59 (2.46, 2.74)
**Comorbidity** (reference: no comorbidity)		
Myocardial infarction	1.18 (0.96, 1.45)	0.88 (0.70, 1.10)
Congestive heart failure	1.58 (1.44, 1.74)	1.20 (1.08, 1.33)
Cerebrovascular disease	1.58 (1.48, 1.70)	1.12 (1.04, 1.22)
Dementia	3.04 (2.67, 3.47)	2.09 (1.80, 2.41)
Renal disease	1.38 (1.21, 1.58)	1.33 (1.15, 1.54)
Paraplegia and hemiplegia	2.21 (1.81, 2.68)	1.46 (1.18, 1.82)
Diabetes mellitus with complication	1.18 (1.11, 1.24)	0.92 (0.86, 0.98)
AIDS/HIV.	1.31 (0.53, 3.25)	1.89 (0.73, 4.93)
**Etiology**		
Hepatitis B virus*	*Reference*	*Reference*
Hepatitis C virus	1.31 (1.22, 1.40)	1.15 (1.07, 1.24)
Other	3.64 (3.51, 3.79)	2.98 (2.86, 3.11)
**Severe liver disease**		
No	*Reference*	*Reference*
Yes	1.54 (1.46, 1.62)	1.56 (1.48, 1.65)
**Income percentile**		
Medical aid beneficiary	1.56 (1.48, 1.65)	1.51 (1.42, 1.60)
≤30	1.20 (1.14, 1.26)	1.20 (1.14, 1.26)
31–70	1.15 (1.10, 1.20)	1.16 (1.11, 1.22)
>70	*Reference*	*Reference*
**Residency area**		
Metropolitan	*Reference*	*Reference*
Rural	1.14 (1.10, 1.18)	1.06 (1.02, 1.10)

* Including patients co-infected with HBV and HCV

**Fig 1:**
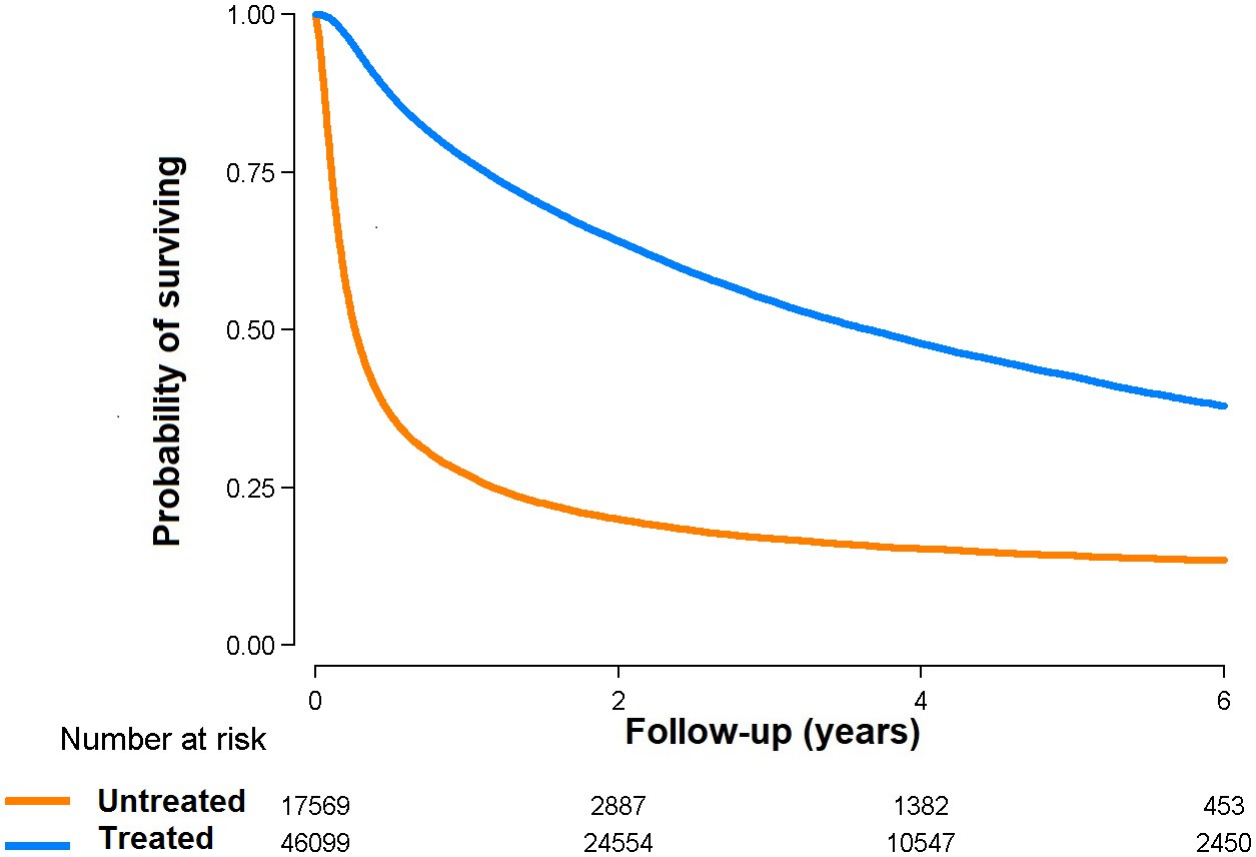
Overall survival from diagnosis of hepatocellular carcinoma by treatment status (N = 63,668).

During 135,123 person-years of follow-up (median follow-up 2.1 years), we observed 37,407 deaths. All-cause mortality rates in the treated and untreated groups were 19.3 and 83.7 per 100 person-years, respectively (Table 3 and Fig 1). Untreated patients had a significantly higher risk of all-cause mortality compared to treated patients even after adjusting for age, sex, year of diagnosis, SEER stage, comorbidity, etiology, severe liver disease, income percentile, and urban vs rural location (fully-adjusted HR comparing untreated vs. treated patients 3.11, 95% CI 3.04–3.18).

**Table 3:** Hazard ratios (95% confidence intervals) for all-cause mortality comparing untreated vs. treated patients with newly diagnosed hepatocellular carcinoma, overall and by SEER stage.

	Mortality rate (per 100 pys)	Crude	Model 1	Model 2
		HR (95% CI)	HR (95% CI)	HR (95% CI)
**Overall**				
**Untreated**	83.75	3.72 (3.64, 3.80)	3.74 (3.56. 3.72)	3.11 (3.04, 3.18)
**Treated**	19.33	*Reference*	*Reference*	*Reference*
**Localized**				
Untreated	40.03	3.15 (3.04, 3.27)	3.10 (2.99. 3.21)	2.94 (2.83, 3.05)
Treated	11.91	*Reference*	*Reference*	*Reference*
**Regional**				
Untreated	168.20	4.04 (3.88, 4.19)	3.90 (3.75. 4.05)	3.71 (3.57, 3.86)
Treated	34.56	*Reference*	*Reference*	*Reference*
**Distant**				
Untreated	289.09	2.78 (2.65, 2.91)	2.65 (2.53, 2.78)	2.49 (2.38, 2.61)
Treated	93.20	*Reference*	*Reference*	*Reference*
**Unknown**				
Untreated	82.78	3.73 (3.54. 3.94)	3.61 (3.42, 3.81)	3.36 (3.19, 3.55)
Treated	19.71	*Reference*	*Reference*	*Reference*

Abbreviations: HR, hazard ratio; CI, confidence interval.

Model 1: Adjusted for sex, age, year of HCC diagnosis, SEER stage, income percentile (Medical aid, ≤ 30^th^, > 30^th^– 70^th^, and > 70^th^), residency area (urban vs rural) and comorbidities; Model 2: Further adjusted for etiology, and severe liver disease.

Among participants who died, 91.7% and 88.9% died due to liver cancer specific cause in the treated and untreated group, respectively. Fully-adjusted HR for liver cancer specific mortality comparing untreated vs. treated patients was 3.05 (95% CI 2.98–3.12) (S1 Table).

**Fig 2:**
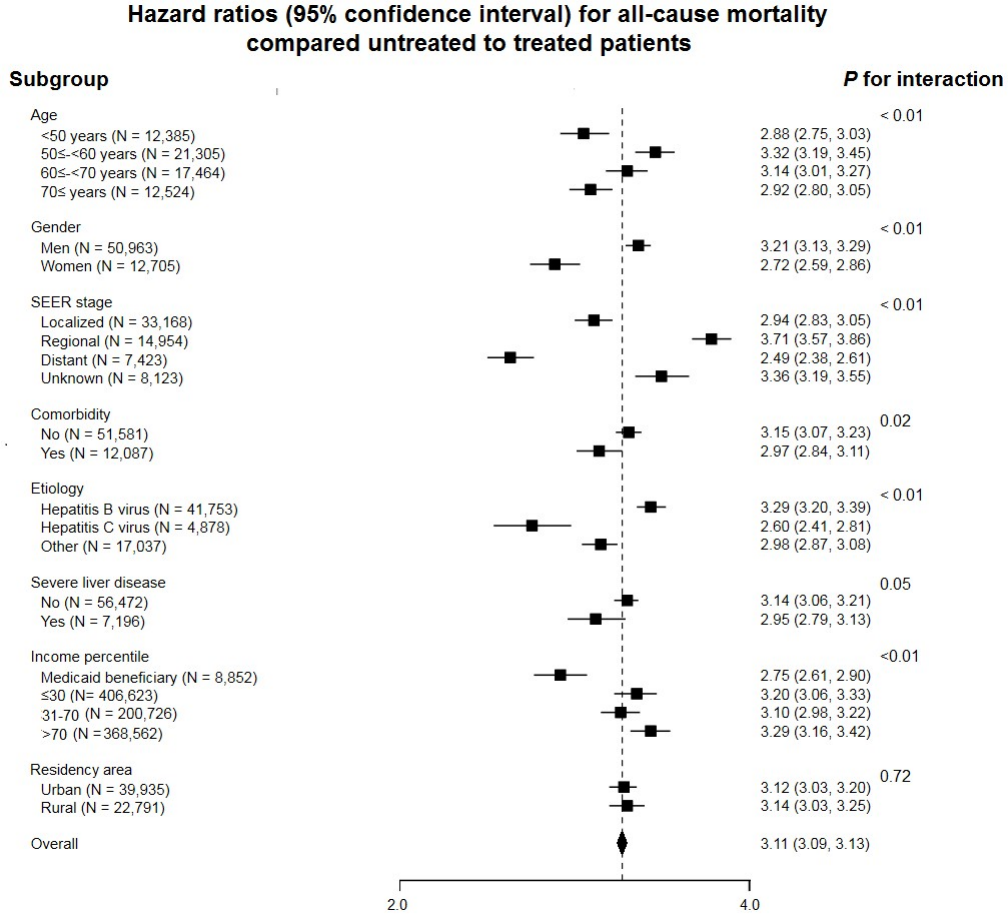
Hazard ratios (95% confidence intervals) for mortality comparing untreated with treated patients with hepatocellular carcinoma in selected subgroups. Adjusted for sex, age, year of HCC diagnosis, SEER stage, income percentile (Medical aid, ≤ 30^th^, > 30^th^– 70^th^, and > 70th), residency area (urban vs rural), comorbidities, etiology, and severity of liver disease.

**Fig 3:**
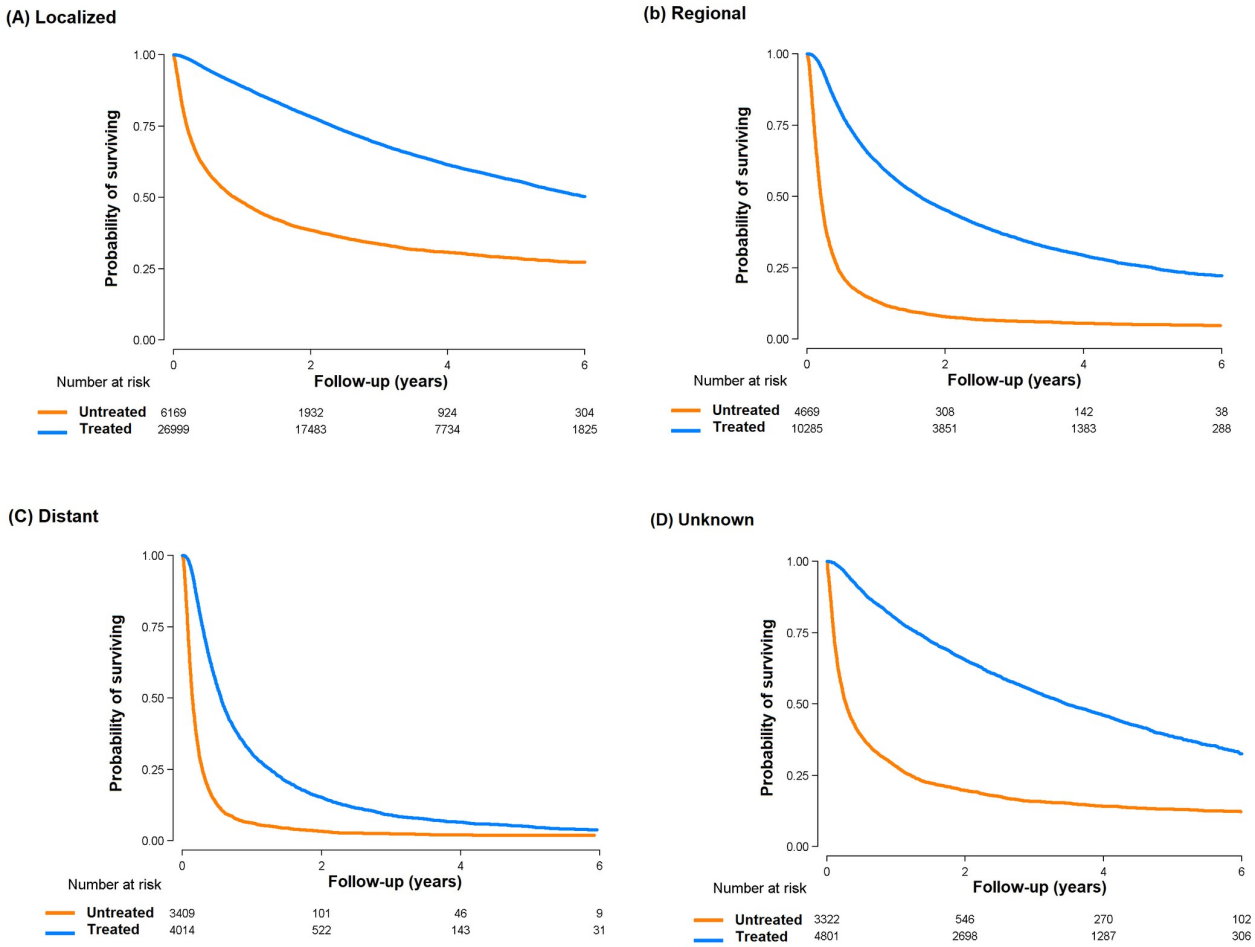
Overall survival from diagnosis of hepatocellular carcinoma by treatment status in subgroups of Surveillance, Epidemiology, and End Results (SEER) stage. (a) Localized disease; (b) Regional disease; (c) Distant disease; (d) Unknown.

In subgroup analysis, the excess mortality in untreated patients was present in all pre-defined subgroups (Fig 2). The association was stronger in men, in patients ≥50 –<70 years of age, and in those with regional disease, without comorbidities and in the highest income category. When the association between treatment and mortality was evaluated by SEER stage (Table 3 and Fig 3), the higher risk of mortality in untreated vs. treated patients was evident in all stages, even in patients with distant disease (HR 2.49, 95% CI 2.38–2.61).

## Discussion

In this large nationwide study, the proportion of untreated HCC patients declined from 2008 to 2013, but still one fourth (24.8%) of newly-diagnosed HCC patients did not receive any HCC-specific treatment in 2013. Older age, advanced SEER stage, severe liver disease, and lower income were associated with being untreated. Untreated patients had a 3-fold higher risk of mortality compared to treated patients. The increased mortality in untreated vs. treated patients was evident in all subgroups, including older age, advanced stage, severe liver disease, and lower income.

The proportion of HCC patients who do not receive specific anticancer treatment varies from 10% to more than 60% across studies [11–13, 21]. Several reasons may explain these differences, including differences in study period, health insurance system, and medical costs. South Korea has a single payer system and all population is covered by the NHIS, explaining the relatively high treatment rates. However, even with a single payer system, we still found differences in treatment status by income category. In the US, patients who were elderly, non-Caucasian, and who had low socioeconomic status, poor liver function, and poor performance status were less likely to receive HCC specific treatment [11, 12, 21]. Consistent with these findings, we also found that elderly patients, those with severe liver disease, and those with lower income less likely to be treated. In addition, female patients had higher risk of not having HCC treatment after controlling confounding factors. It was similar with previous literature that gender inequities manifests itself in both lower health investments as well as worse health status of women relative to men [22].

Stage at diagnosis was also significantly associated with treatment status [11]. Advanced stage HCC was associated both with lower likelihood of receiving treatment and with poor survival [4]. Diagnosing HCC at early stages using surveillance may thus lead to increased likelihood of receiving treatment and of improving HCC survival. Indeed, HCC surveillance has been linked to earlier stage diagnoses and improved survival [23–25], and implementation of a national cancer surveillance program in South Korea was proposed as an explanation of HCC prognosis improvement over the last 20 years [26]. Nevertheless, even with a national surveillance program in Korea, a country with relatively high prevalence of hepatitis B virus infection and high risk of HCC, adherence to regular HCC surveillance remains poor [27, 28]. Intensifying population HCC surveillance efforts in high-risk populations could increase treatment rates and ultimately improve survival of HCC patients.

A relatively high proportion of patients with early stage disease at diagnosis also do not receive HCC-specific treatment (in our study, 18.6% of patients with localized SEER stage at diagnosis did not receive treatment) [21]. Since we used an administrative claims database, we did not have detailed information on the reasons why these patients did not receive treatment. Hence, future studies need to identify reasons for withholding treatment as well as barriers to HCC treatment in these patients.

In our study, untreated patients showed a 3-fold higher risk of mortality compared to treated patients, and the association between treatment and mortality was observed in all predefined subgroups including older patients, distant SEER stage, and severe liver disease. Active HCC therapy is associated with better outcomes [11], including in old patients, in patients with extrahepatic metastasis, and in patients with decreased liver function [29–32]. As a consequence, increasing treatment rates may benefit these subgroups as well. A multidisciplinary approach may help guide treatment in these difficult-to-treat populations [33].

Our data has several limitations. Although HCC-treatment improves outcomes, the difference in mortality between treated and untreated patients in our study cannot be only attributed to treatment effects. Since we used an administrative claims data, important prognostic information that might be associated with treatment and survival, such as functional status, and reason for no treatment was not available. However, when we conducted subgroup analysis in all predefined subgroups including older patients, distant SEER stage, and severe liver disease, the results were similar in all subgroup. Thus, non-treatment affects to the clinical outcomes in HCC in any reason. Thus, non-treatment affects to the clinical outcomes in HCC in any reason. Also, as this study was performed in South Korea, which is one of the most ethnically homogeneous countries that has a unique single payer system, generalizability of the study findings to other population at different healthcare system requires further evaluation.

## Conclusions

In summary, untreated HCC is still common, even among patients diagnosed at early stage. Untreated HCC patients had a higher risk of mortality compared to treated patients. Older age, advanced stage, severe liver disease and lower income was associated with a lower likelihood of receiving HCC treatment. The proportion of untreated HCC patients declined progressively from 2008 to 2013 in South Korea, but almost one fourth newly-diagnosed patients did not receive HCC-specific treatment in 2013. Efforts to identify HCC cases at an early stage, to identify and remove barriers for HCC treatment, and to improve treatment rates could continue the trend towards improved HCC survival.

## Supporting information

S1 Table Hazard ratios (95% confidence intervals) for liver cancer specific mortality comparing untreated vs. treated patients with newly diagnosed hepatocellular carcinoma, overall and by SEER stage. (DOCX)
