# Supplementary material for: Survival in untreated hepatocellular carcinoma: A national cohort study
Source: PLoS One. 2021 Feb 4;16(2):e0246143. doi: 10.1371/journal.pone.0246143 (PMC7861368; doi:10.1371/journal.pone.0246143)
Supplement: S1 Table — (DOCX) [file pone.0246143.s001.docx]

**S1 Table.** Hazard ratios (95% confidence intervals) for liver cancer specific mortality comparing untreated vs. treated patients with newly diagnosed hepatocellular carcinoma, overall and by SEER stage.

|  | **Mortality rate**  **(per 100 pys)** | **Crude**  **HR (95% CI)** | **Model 1**  **HR (95% CI)** | **Model 2**  **HR (95% CI)** |
| --- | --- | --- | --- | --- |
| **Overall** |  |  |  |  |
| **Untreated** | 74.45 | 3.59 (3.51, 3.67) | 3.54 (3.47. 3.63) | 3.05 (2.98. 3.12) |
| **Treated** | 17.72 | *Reference* | *Reference* | *Reference* |
|  |  |  |  |  |
| **Localized** |  |  |  |  |
| Untreated | 33.07 | 2.96 (2.85, 3.08) | 2.93 (2.81. 3.05) | 2.81 (2.70. 2.92) |
| Treated | 10.46 | *Reference* | *Reference* | *Reference* |
| **Regional** |  |  |  |  |
| Untreated | 156.83 | 3.96 (3.81, 4.12) | 3.87 (3.72. 4.03) | 3.72 (3.57. 3.87) |
| Treated | 32.73 | *Reference* | *Reference* | *Reference* |
| **Distant** |  |  |  |  |
| Untreated | 270.27 | 2.68 (2.55, 2.81) | 2.60 (2.48, 2.73) | 2.47 (2.35, 2.59) |
| Treated | 90.50 | *Reference* | *Reference* | *Reference* |
| **Unknown** |  |  |  |  |
| Untreated | 71.18 | 3.54 (3.34. 3.75) | 3.46 (3.27, 3.66) | 3.26 (3.08, 3.46) |
| Treated | 17.81 | *Reference* | *Reference* | *Reference* |

Abbreviations: HR, hazard ratio; CI, confidence interval.

Model 1: Adjusted for sex, age, year of HCC diagnosis, SEER stage, income percentile (Medical aid , ≤ 30^th^, > 30^th^ – 70^th^, and > 70^th^), residency area (urban vs rural) and comorbidities; Model 2: Further adjusted for etiology, and severe liver disease .
